# Health sector involvement in the management of female genital mutilation/cutting in 30 countries

**DOI:** 10.1186/s12913-018-3033-x

**Published:** 2018-04-04

**Authors:** R. Elise B. Johansen, Mai Mahgoub Ziyada, Bettina Shell-Duncan, Adriana Marcusàn Kaplan, Els Leye

**Affiliations:** 1 Norwegian Centre for Violence and Traumatic Stress Studies, PB: 181 Nydalen, 0409 Oslo, Norway; 2 Department of Anthropology, University of Washington, M230 Denny Hall, Box 353100, Seattle, WA 98195-3100 USA; 3 Wassu-UAB Foundation, Universitat Autònoma de Barcelona, Módul de Recerca A - Campus Bellaterra, 08193 Barcelona, Spain; 4 International Centre for Reproductive Health, Ghent University, De Pintelaan 185 UZP114, 9000 Ghent, Belgium

**Keywords:** Female genital mutilation/cutting, Female circumcision, Health policy, Healthcare, Prevention, Countries of migration, Countries of origin

## Abstract

**Background:**

For the last decades, the international community has emphasised the importance of a multisectoral approach to tackle female genital mutilation (FGM/C). While considerable improvement concerning legislations and community involvement is reported, little is known about the involvement of the health sector.

**Method:**

A mixed methods approach was employed to map the involvement of the health sector in the management of FGM/C both in countries where FGM/C is a traditional practice (countries of origin), and countries where FGM/C is practiced mainly by migrant populations (countries of migration). Data was collected in 2016 using a pilot-tested questionnaire from 30 countries (11 countries of origin and 19 countries of migration). In 2017, interviews were conducted to check for data accuracy and to request relevant explanations. Qualitative data was used to elucidate the quantitative data.

**Results:**

A total of 24 countries had a policy on FGM/C, of which 19 had assigned coordination bodies and 20 had partially or fully implemented the plans. Nevertheless, allocation of funding and incorporation of monitoring and evaluation systems was lacking in 11 and 13 of these countries respectively. The level of the health sectors’ involvement varied considerably across and within countries. Systematic training of healthcare providers (HCP) was more prevalent in countries of origin, whereas involvement of HCP in the prevention of FGM/C was more prevalent in countries of migration. Most countries reported to forbid HCP from conducting FGM/C on both minors and adults, but not consistently forbidding re-infibulation. Availability of healthcare services for girls and women with FGM/C related complications also varied between countries dependent on the type of services. Deinfibulation was available in almost all countries, while clitoral reconstruction and psychological and sexual counselling were available predominantly in countries of migration and then in less than half the countries. Finally, systematic recording of FGM/C in medical records was completely lacking in countries of origin and very limited in countries of migration.

**Conclusion:**

Substantial progress has been made in the involvement of the health sector in both the treatment and prevention of FGM/C. Still, there are several areas in need for improvement, particularly monitoring and evaluation.

**Electronic supplementary material:**

The online version of this article (10.1186/s12913-018-3033-x) contains supplementary material, which is available to authorized users.

## Background

Female genital mutilation/cutting (FGM/C) is a global health issue. More than 200 million girls and women in 30 countries in Africa, Asia and the Middle East have undergone the practice [1], and more than 3.6 million girls are annually at risk [2]. The impact of FGM/C is also spreading further through migration to other parts of the world including Europe [3–6], the USA [7, 8], Australia [9] and Canada [10]. Hence policies are needed to address this issue in both countries of origin and countries of migration.

FGM/C encompasses all procedures that involve the partial or total removal of external genitalia or other injury to the female genital organs for non-medical reasons [11], and is most commonly performed on minors [12]. The World Health Organization (WHO) classifies FGM/C into four types according to the type of tissue removed: Type I partial or total removal of the clitoris (clitoridectomy) and/or the prepuce, Type II partial or total removal of the clitoris and the labia minora and/or labia majora (excision), Type III narrowing of the vaginal orifice with the creation of a covering seal by cutting and appositioning the labia majora and/or minora, with or without excision of the clitoris (infibulation). Type IV includes all other harmful procedures to the female genitalia for non-medical purposes, for example pricking, piercing, incising, scraping and cauterization.

FGM/C is associated with a greater risk for a series of health complications, dependent on the extent and type of tissue removed. Immediate health risks include pain, haemorrhage, infection, urinary retention and injury to the urethra, wound healing problems and death [13]. Long-term health complications include genitourinary (urinary tract infection, bacterial vaginosis, problems with menstruation) [14], obstetrical (caesarean section, postpartum haemorrhage, episiotomy, prolonged labour, tears or lacerations, instrumental delivery, difficult labour, external maternal hospital stay, still birth and early neonatal death, infant resuscitation at delivery) [15], sexual (dyspareunia, no sexual desire and reduced sexual satisfaction) [16], and psychological (post-traumatic stress disorder, anxiety disorder and depression) consequences [17]. FGM/C, in particular infibulation, can also interfere with other medical procedures such as gynaecological examination, cytology testing, post-abortion evacuation of the uterus and insertion of intrauterine device [18].

Considering the health impact of FGM/C, the World Health Organization (WHO) has played a key role in tackling the issue from its first international conference on FGM/C in 1979 onwards [19]. The WHO also issued the first United Nations (UN) policy statement on FGM/C in 1997 [20], reiterating it in 2008 [11]. The statement emphasized the importance of broad-based, long-term commitment as well as a multisectoral approach involving education, finance, justice, women’s affairs and health. In 2001, the WHO developed its first policy guidelines for the health sector [21], accompanied by practical and clinical guidelines [22–24] that were updated in 2016 [18]. The UN emphasize and outline the role of healthcare providers (HCP) in primary prevention and provision of care [11].

In 2008 and 2012 the World Health Assembly (WHA) and the UN General Assembly respectively agreed on resolutions against FGM/C urging all member states to develop, support and implement national policies and action plans as well as to allocate sufficient resources for its implementation [25, 26]. The resolutions also highlighted the importance of incorporating clear targets and indicators in the national plans and policies for the effective monitoring, impact assessment and coordination of programmes. The WHA also commits its member states to follow up and regularly report on a set of points targeting prevention (in particular community based interventions), legislation, guidelines and provision of care [26].

Literature on FGM/C shows that several countries have developed national policies [27–32], guidelines [33–40] and legislations [41–43], as well as making progress in the involvement of community based interventions [43–46]. Less is known however about the involvement of the health sector in national plans, nor the implementation levels, allocated resources, coordination and monitoring and evaluation of such plans in the different countries. A 2012 review of policies in 28 countries in the European Union (EU) reports insufficient and unequal distribution of support- and health services as well as inconsistent funding to ensure access to services [32]. No similar data is available from non-EU countries with high numbers of migrants from FGM/C practicing countries, nor from countries where FGM/C is traditionally practiced. Nevertheless, a 2010 progress report on the WHA resolution from African member states highlight the involvement of the health sector as an area in need for improvement [43].

In this paper, we aspire to fill this knowledge gap by presenting an overview of the current status of the involvement of the health sector in national plans on FGM/C in 30 countries, 11 of which are countries where FGM/C is a traditional practice (hereafter referred to as countries of origin), and 19 countries where FGM/C is practiced mainly by migrant populations (hereafter referred to as countries of migration). We present the data unaggregated to highlight areas for improvement for each country and to facilitate future measurement of progress.

## Method

To identify elements central to the health sector involvement in national policies on FGM/C, we first conducted a desktop review of the WHO policies and declarations [11, 26, 47, 48], national and clinical guidelines [18, 21, 27–31, 33–37, 49] and other relevant literature [8, 35, 38, 39, 41, 50]. The key elements identified were: Training of HCP, involving HCP in the preventive work, legal regulations on HCP duties to avert and report as well as to perform FGM/C, and finally the availability of healthcare services (deinfibulation, clitoral reconstruction, sexual and psychological counselling). These elements were then grouped in three categories: 1) training and duties of HCP, 2) performing FGM/C by HCP, and 3) availability of health services. A fourth category ‘the availability and systematic use of medical codes’ was then added since medical records are important not only for proper medical care, but also to inform health policies. REBJ and MMZ used these categories, together with questions on the existence and the level of implementation of national policies on FGM/C, allocation of funds, coordination, and monitoring and evaluation systems to develop a questionnaire with closed- and open-ended questions. EL and BSD commented and revised the questionnaire. The questionnaire was then pilot-tested in three countries resulting in minor revisions. The final version (Additional file 1) was then distributed through e-mails in the period April–June 2016 to 61 countries, of which 33 were countries of origin and 29 countries of migration.^1^ For data collection in the 33 countries of origin, assistance was first asked from the WHO headquarter and the UNFPA-UNICEF Joint Programme on Female Genital Mutilation, but they were unable to help at the time. Therefore, we have instead contacted the WHO country offices, NGOs and researchers known to work on FGM/C in these countries inviting them to participate in the study or to put us in contact with the relevant persons/authorities. Up to three reminder e-mails were sent to non-respondents. Eleven respondents representing 11 countries of origin returned filled questionnaires. These respondents filled the questionnaires in their capacity as: the WHO country office (Ghana), researchers (Burkina Faso, the Gambia, Iran, Kenya, Mali, Sierra Leone and Sudan) and NGO’s (Egypt, Ethiopia and Somaliland). Supportive documents were provided for only two countries (Iran and the Gambia).

Invitations to participate were also e-mailed to government representatives, researchers and NGOs known to work on FGM/C in 29 countries of migration. Up to three reminder e-mails were sent to non-respondents. Twenty-one respondents representing 19 countries of migration returned filled questionnaires. Fifteen of the respondents were researchers representing 14 countries (Australia, Austria, Belgium, Finland, Ireland, Italy, the Netherlands, Norway, Portugal, Saudi-Arabia, Spain, Sweden, UK and the USA), as UK had two responding researchers. Three of the respondents were government representatives for three countries (Greece, Slovakia and Sweden). In one country (Switzerland) the questionnaire was filled by a researcher/WHO. Finally, two NGO’s representatives filled the questionnaire for two countries (France and Germany). Supportive documents/sources were also provided for ten countries (Australia, Belgium, Greece, Ireland, the Netherlands, Norway, Portugal, Slovakia, Spain and Sweden). Since both the UK and Sweden had more than one respondent each with conflicting response, we decided after cross-checking with provided/available sources which response to use.

The data was entered into SPSS 22 and analysed. We then cross-checked the results from each country with provided or available sources and documents. Inconsistencies in certain data (duty to avert and duty to report, availability of sexual and psychological counselling services, availability and systematic use of medical codes and legal regulations for HCP concerning performing FGM/C on adults) were discovered. Therefore we contacted all respondents in 2017 and through face-to-face and/or phone interviews and e-mail cross-checked, clarified potential misunderstandings and largely corroborated all information with a special focus on areas of discrepancy. The corroborated quantitative data was then reanalysed and presented in tables, while clarifying and additional provided data were used to elucidate the different contexts and practices in different countries.

A limitation of the study is that the focus on national policies obscured in-country variation. Another methodological limitation is the low response rate, particularly in countries of origin. Thus, the findings may not be representative, but rather biased towards countries that have FGM/C high on their political agenda. Furthermore, the low level of responses from responsible government departments can be a weakness, as some of the respondents might have lacked a full knowledge of government policy. Still, having most questionnaires filled by non-governmental representatives could also give more realistic and unbiased information.

## Results

### National health policies and their implementation

As shown in Table 1, 24 out of 30 countries had a policy on FGM/C in the form of a national plan of action or national or professional guidelines. These policies were fully or partially implemented in 20 countries and not implemented in four (Gambia, Somaliland, Spain and Sweden). Funding for implementation was allocated in 13 of the 24 countries (six countries of origin and seven countries of migration), while seven countries managed to have some degree of implementation without allocated governmental funding. Many countries (19/24) had assigned coordinating bodies for the implementation of the plans. The weakest point was a scarcity in systems for monitoring and evaluation which was in place in only 11 countries, four in countries of origin and seven in countries of migration. Overall, eight of the countries were ahead of the others in regard to having policies on FGM/C that were implemented, funded, coordinated and with systems for monitoring and evaluation in place. These included four countries of origin (Burkina Faso, Kenya, Mali and Sudan) and four countries of migration (France, Ireland, the Netherlands and Norway).

**Table 1:** Overview of countries that have national policies on FGM/C, allocated funding, coordination, monitoring and evaluation systems

	Have national policy on FGM/C	Fully or partially implemented	Have allocated budget	Coordination bodies are assigned	Monitoring and evaluation systems in place
Countries of origin					
Burkina Faso	√	√	√	√	√
Egypt	√	√	√		
Ethiopia	√	√	√	√	
Gambia, The	√			√	
Ghana	√	√		√	
Iran					
Kenya	√	√	√	√	√
Mali	√	√	√	√	√
Sierra Leone					
Somaliland	√				
Sudan	√	√	√	√	√
*Sub total*	*9*	*7*	*6*	*7*	*4*
Countries of migration					
Australia	√	√			
Austria					
Belgium	√	√		√	
Finland	√	√		√	√
France	√	√	√	√	√
Germany	√	√		√	
Greece					
Ireland	√	√	√	√	√
Italy	√	√	√	√	
Netherlands, The	√	√	√	√	√
Norway	√	√	√	√	√
Portugal	√	√	√	√	
Saudi Arabia					
Slovakia					
Spain	√			√	√
Sweden	√				
Switzerland	√	√	√	√	
UK	√	√		√	√
USA	√	√			
*Sub total*	*15*	*13*	*7*	*12*	*7*
*Total*	*24*	*20*	*13*	*19*	*11*

### Training and duties of healthcare providers

Table 2 presents an overview of countries where HCP: 1) receive systematic or ad-hoc training on FGM/C, 2) have a duty to inform patients on the harmful effects of FGM/C, 3) have a duty to avert when there is a risk of FGM/C, and 4) have a duty to report when FGM/C has already taken place. Duty to avert is defined as the HCP duty to make an active effort to dissuade families from subjecting their daughters to FGM/C and/or to report to the child protection or the police whenever there is a high possibility that a child is at imminent risk of being subjected to FGM/C. Duty to report, on the other hand is defined as the HCP duty to report to child protection and/or the police all suspected or confirmed cases where FGM/C has taken place and is considered illegal.

**Table 2:** Countries where healthcare providers receive training on FGM/C and have duties to health educate patients, avert and report FGM/C

	Training for HCP on FGM/C	Duty to educate patients on FGM/C	Legal duty to avert cases of planned FGM/C	Legal duty to report cases of performed FGM/C
Countries of origin				
Burkina Faso	√			
Egypt	√^Ad-hoc^			
Ethiopia	√		√	
Gambia, The	√		√	√
Ghana	√			
Iran				
Kenya	√		√	√
Mali	√			
Sierra Leone	√^Ad-hoc^			
Somaliland	√			
Sudan	√			
*Sub total*	*10*	*0*	*3*	*2*
Countries of migration				
Australia	√^Ad-hoc^		√	
Austria	√			√
Belgium	√^Ad-hoc^		√	
Finland	√	√	√	√
France	√	√		√
Germany			√	
Greece			√	√
Ireland	√^Ad-hoc^		√	√
Italy	√	√	√	√
Netherlands, The	√	√	√	√
Norway	√^Ad-hoc^	√	√	
Portugal	√	√	√	√
Saudi Arabia				
Slovakia			√	√
Spain	√^Ad-hoc^	√	√	√
Sweden	√^Ad-hoc^		√	√
Switzerland			√	√
UK	√		√	√
USA	√^Ad-hoc^	√	√	√
*Sub total*	*14*	*8*	*16*	*14*
*Total*	*24*	*8*	*19*	*16*

Training of HCP on FGM/C was carried out in 24 out of the responding countries (10 countries of origin and 14 countries of migration). Among the 10 countries of origin, the training was reported to be systematic in eight, as FGM/C is included in one or more curricula for HCP. In countries of migration only half of the 14 countries have systematic training, whereas the other half conducts it on an ad-hoc basis.

HCP have a duty to provide health education on FGM/C to their patients in only eight of the responding countries, all of which are countries of migration. A duty to avert was reported from 19 countries, three countries of origin (Ethiopia, Gambia and Kenya) and 16 countries of migration. Duty to report was present in 16 countries, 14 countries of migration and only two countries of origin (Gambia and Kenya).

In several countries the duties to avert and report FGM/C differ between HCP and other public servants as HCP are bound by medical confidentiality. Some countries have specifically exempted HCP from medical confidentiality in regards to either the duty to avert or the duty to report or both. Concerning duty to avert, HCP in Germany should first try to discourage the family from performing FGM/C through counselling, and only report to the police if they have concrete information of a planned FGM/C. In Norway and Australia, HCP also have a duty to avert, but the threshold to reporting to child protection services is set lower than in Germany. Imminent risk is required for reporting to the Norwegian and Australian police but not to the child protection services. In the Netherlands, only doctors have the authority to report concrete cases of risk to the child protection services, thus other HCP have to involve doctors in such cases. In three countries of origin medical confidentiality was said to hinder duty to avert (Burkina Faso, Egypt and Mali).

Regulations concerning reporting cases where a girl or woman has already been subjected to FGM/C is similarly diverse. In Austria and the Netherlands, HCP have a duty to register all cases of FGM/C, but only report those believed to be illegal to child protection services or the police. Illegal cases of FGM/C in these two countries are all cases that occurred after settlement, regardless of age. In contrast, illegal cases of FGM/C in Ireland cover only girls under 18. Therefore the duty to report performed FGM/C in Ireland only applies for minors. In Australia, HCP do not have a general duty to report FGM/C but there are some exceptions for minors, and HCP are advised to contact the legal and legislative services for guidance. In five countries with national laws against FGM/C, medical confidentiality was said to prevent reporting (Burkina Faso, Egypt, Ethiopia, Ghana and Mali).

Repercussions for HCP for not fulfilling their eventual duties to health educate, avert or report vary from no repercussions (e.g. USA), fines (e.g. Gambia), disciplinary sanctions (e.g. Austria) to imprisonment (e.g. Norway).

### Legal regulations on healthcare providers’ performance of FGM/C

The trend of replacing traditional circumcisers with HCP in performing FGM/C (medicalization of FGM/C) [51] has long been a source of concern for the UN and many professional organizations, including the International Federation of Gynecology and Obstetrics (FIGO) [47].

While mapping legal regulations on medicalization of FGM/C (see Table 3), we have distinguished between performing FGM/C on minors and consenting adults, and between primary FGM/C and re-infibulation. Reinfibulation refers to the restoration of infibulation after it has been opened, commonly during childbirth, whereas primary FGM/C refers to the original procedure. In most countries, HCP are legally prohibited from performing FGM/C on minors. This included six countries of origin and 18 countries of migration. Primary FGM/C by HCP is also illegal on adults in these six countries of origin, but only in 16 of the countries of migration as the law in USA and Portugal covers minors only. Although the law in Ireland also targets minors only, it is still illegal for HCP to perform FGM/C on adults. Reinfibulation by HCP on the other hand, is prohibited in only 16 countries, three of which are countries of origin. This leaves 14 countries without legal regulation on reinfibulation, including Sudan, a country where reinfibulation is a common practice [52–54]. Six out of these 14 countries also have no legal regulation on FGM/C at the national level, including five countries of origin (Iran, Mali, Sierra Leone, Somaliland and Sudan) and one country of migration (Saudi Arabia).

**Table 3:** Countries where it is illegal for healthcare providers to perform FGM/C

	On Minors	On Adults	Re-infibulation
Co4untries of origin			
Burkina Faso	√	√	√
Egypt	√	√	
Ethiopia	√	√	
Gambia, The	√	√	
Ghana	√	√	√
Iran			
Kenya	√	√	√
Mali			
Sierra Leone			
Somaliland			
Sudan			
*Sub total*	*6*	*6*	*3*
Countries of migration			
Australia	√	√	√
Austria	√	√	√
Belgium	√	√	
Finland	√	√	√
France	√	√	√
Germany	√	√	√
Greece	√	√	
Ireland	√	√	√
Italy	√	√	√
Netherlands, The	√	√	√
Norway	√	√	√
Portugal	√		√
Saudi Arabia			
Slovakia	√	√	√
Spain	√	√	
Sweden	√	√	√
Switzerland	√	√	
UK	√	√	√
USA	√		
*Sub total*	*18*	*16*	*13*
*Total*	*24*	*22*	*16*

### Availability of healthcare services for girls and women subjected to FGM/C

Table 4 shows data on the availability of two surgical procedures (deinfibulation and clitoral reconstruction), and two types of counselling (psychological and sexual) to girls and women who have already been subjected to FGM/C.

**Table 4:** Countries with available medical services for women with FGM/C

	Deinfibulation	Psychological counselling	Sexual counselling	Clitoral re-construction
Countries of origin				
Burkina Faso	√	√	√	√
Egypt	√			√^Private^
Ethiopia	√			
Gambia, The	√			
Ghana				
Iran				
Kenya	√	√	√	
Mali	√			√^Private^
Sierra Leone				
Somaliland	√			
Sudan	√			
*Sub total*	*8*	*2*	*2*	*3*
Countries of migration				
Australia	√			√
Austria	√	√	√	√^Private^
Belgium	√	√	√	√
Finland	√			
France	√	√	√	√
Germany	√			√^Private^
Greece	√	√	√	
Ireland	√	√	√	
Italy	√	√	√	√
Netherlands, The	√	√	√	√^Private^
Norway	√			
Portugal	√			
Saudi Arabia	√			
Slovakia	√	√	√	
Spain	V			√
Sweden	√	√	√	√
Switzerland	√	√	√	√
UK	√	√	√	
USA	√			
*Sub total*	*18*	*11*	*11*	*10*
*Total*	*26*	*13*	*13*	*13*

Deinfibulation is a minor surgical procedure carried out to reopen the vaginal introitus in girls and women who have undergone infibulation [39], so as to ease many of the complications and risks associated with infibulation [18, 22, 55] and to facilitate childbirth.

Clitoral reconstruction following FGM/C is a surgical technique directed toward women who have undergone any type of FGM/C that involve the cutting of the clitoris [56]. It is reported to reduce clitoral pain, improve sexual pleasure, and restore a vulvar appearance similar to that of women not subjected to FGM/C [56–58]. However, since evidence on its benefits, safety and efficacy is inconclusive, the WHO is apprehensive to recommending clitoral reconstruction as a best practice [18].

In contrast, the WHO guidelines recommend cognitive behavioural therapy for women experiencing symptoms consistent with anxiety disorders, depression or post-traumatic stress. The WHO also suggests that both psychological support and sexual counselling should be available in relation to surgical interventions such as clitoral reconstruction and deinfibulation [18].

Deinfibulation services are available in a total of 27 countries (eight countries of origin and all of the 19 countries of migration). In the three countries of origin where deinfibulation services are unavailable, infibulation is uncommon. Nevertheless, in a few countries (e.g. Slovakia and Switzerland), deinfibulation services are only available at private clinics, though the cost can be refunded by public health insurance. Moreover, in Spain and Portugal, deinfibulation is mainly available in the context of pregnancy and childbirth.

Clitoral reconstruction is available in 13 countries, of which three are countries of origin and the other 10 are countries of migration. Nevertheless, in only one country of origin (Burkina Faso) and seven countries of migration is clitoral reconstruction provided through public health services.

Specialized psychological and sexual counselling services on FGM/C are available in 13 countries, including only two countries of origin (Burkina Faso and Kenya). Some countries reported good national coverage, while others reported significant regional variation. This variation was attributed to variation in competence on FGM/C among psychologists, psychiatrists and sexologists. In France, psychological and sexual counselling is reported to be widely available since all relevant education programs for HCP have a special module on FGM/C. The Netherlands also reported good coverage with nine specialized centres on FGM/C offering psychological and sexual counselling. Austria, Ireland, Switzerland and the UK reported significant variation in the availability of specialized services between regions, with most services concentrated in big cities. In two countries (Belgium and Sweden), counselling was primarily offered in relation to requests for clitoral reconstruction. In seven countries of migration (Australia, Finland, Germany, Norway, Portugal, Spain and the USA), there are no available services with special competence on FGM/C and patients are referred to general services.

### Medical registration and documentation

As shown in Table 5, only 13 countries reported to have a designated medical code on FGM/C, none of which are countries of origin. Furthermore, only two countries (the UK and the Netherlands) reported systematic use of these codes.

**Table 5:** Countries where codes on FGM/C are available and used systematically in medical records

	Available	Used sytematically^a^
Countries of origin		
Burkina Faso		
Egypt		
Ethiopia		
Gambia, The		
Ghana		
Iran		
Kenya		
Mali		
Sierra Leone		
Somaliland		
Sudan		
*Sub total*	*0*	*0*
Countries of migration		
Australia	√	
Austria		
Belgium	√	
Finland		
France	√	
Germany	√	
Greece		
Ireland	√	
Italy	√	
Netherlands, The	√	√
Norway	√	
Portugal	√	
Saudi Arabia		
Slovakia		
Spain	√	
Sweden	√	
Switzerland		
UK	√	√
USA	√	
*Sub total*	*13*	*2*
*Total*	*13*	*2*

^a^Codes for FGM/C are used consistently in conformance with established guidelines and routines

Six of the countries that reported to have no designated medical code for FGM/C (Austria, Burkina-Faso, Ghana, Kenya, Slovakia and Switzerland), reported to use the 10th version of the International Classification of Diseases (ICD) coding system [59].

At the time of data-collection Finland did not have a designated medical code on FGM/C. Upon cross-checking of data however, it was reported that Finland introduced a designated code on FGM/C in 2017 to be used systematically in birth- and antenatal registries.

## Discussion

National policies on FGM/C can signify governmental commitment to the prevention and provision of care for those at risk of FGM/C and those who have been subjected to FGM/C, and might be triggered by the ratification of certain legally binding policy documents, such as the Maputo Protocol or the Istanbul Convention. National action plans, in particular, provide a platform and structure for long-term and systematic work on FGM/C whereby different sectors can collaborate effectively. To enable monitoring and evaluation, and accountability, distribution of responsibilities, targets and outcome indicators should be clearly stated. Hence, significant efforts have been made in securing that both countries of origin and countries of migration have such policies in place [32, 60]. Twenty-four of the participating countries in this study reported to have a national policy on FGM/C, of which 19 countries had clear distribution of responsibilities (Table 1). Nevertheless, 13 countries did not allocate funding for their plans and only few established systems for monitoring and evaluation. Moreover, the extent of involvement of the health sector in these plans varied considerably across the participating countries.

HCP can play a key role in the prevention of FGM/C by providing health education to patients and/or parents during consultations, as their educational background and social status give extra credit to their messages [11, 37, 44]. Also, the regular interactions with families provide them with unique opportunities to share such information. Our findings show that providing health education on FGM/C to patients and/or parents during consultations is part of HCP duties in only eight countries, all of which are countries of migration (Table 2). It is striking to see that the preventive role of HCP is highly underused in countries of origin. Several studies have identified numerous challenges to the involvement of HCP, particularly in countries of origin. Some HCP support FGM/C or consider it as a sensitive issue and consequently resist working against the practice [44, 61–64]. This resistance is further aggravated by a high workload [44] and the lack of skills to adequately address FGM/C [65].

On the other hand, as shown in Table 2 many countries oblige HCP to avert (three countries of origin and 16 countries of migration) and/or report FGM/C (two countries of origin and 14 countries of migration), duties that unlike provision of health education may present ethical challenges for HCP [66]. One particular challenge is the problem of dual loyalty where HCP have the simultaneous obligation to a patient and to a third party, often the state [67]. Medical ethics have long recognized the paramount importance of loyalty to patients in the form of confidentiality and the patient’s best interest. Increasingly, health professionals are asked to weigh this loyalty against objectives of governments or other third parties. Dual loyalty poses a particular challenge when patient’s confidentiality poses a risk of a human rights violation, such as in the case of FGM/C. Where state regulations codify “duty to avert FGM/C” or “duty to report FGM/C”, the problem of dual loyalty of health care providers must be addressed. Numerous countries exempt HCP from medical confidentialities in cases of violence against children. In such cases, either the risk and/or the occurrence of FGM/C are defined as a serious abuse, thus the child-protection laws allow or compel HCP to break medical confidentiality in the best interest of the child [32]. There is however disagreement as to whether reporting is in the best interest of the child given that FGM/C is not the same as repetitive, recurrent child abuse [32]. If reporting leads to a care order for the girl in question or imprisonment of her parents, some see this as compromising the child’s best interest, as she loses the care of her parents in addition to being subjected to FGM/C [68–70]. On the other hand, the reporting of parents might protect other female siblings from being subjected to FGM/C. Another concern is a fear that mandatory reporting can deter parents from seeking necessary healthcare for their children for fear of legal repercussions [71].

Another ethical dilemma for HCP is requests to perform FGM/C. In some countries HCP are replacing traditional circumcisers in performing FGM/C as a way of harm reduction [51, 72]. In Sudan, midwifes were trained to perform FGM/C [73] and in Egypt FGM/C was allowed only in State hospitals “by trained physicians under hygienic conditions” [62]. A recent study found that 26% of women age 15 to 49 with FGM/C had been cut by a HCP, with the highest rates in Sudan (67%), Egypt (38%), Guinea (15%), Kenya (15%) and Nigeria (13%) [51].

The WHO, however, condemns the medicalization as it is considered a perpetuation and legitimization of a harmful practice that counteracts efforts towards its abandonment [18, 47, 51, 54, 59, 72, 74]. Moreover, HCP also perform FGM/C for reasons other than harm reduction, such as financial gain, support for the practice and pressure from the communities [75]. As shown in Table 3, HCP are prohibited from performing FGM/C on minors in 24 countries (six countries of origin and 18 countries of migration) and on adults in 22 countries (six countries of origin and 16 countries of migration), including two countries (Egypt and Kenya) with high FGM/C medicalization rates. Sudan, on the other hand, has yet to regulate the problem of medicalization in the country. Furthermore, eight of the countries that prohibit HCP from performing FGM/C do not specifically stipulate reinfibulation as illegal in their laws (Table 3). This could be problematic since reinfibulation is not clearly stated in any of the four types of FGM/C in the WHO classification, and prohibiting FGM/C per se, might not always be interpreted to include reinfibulation [76]. Another problematic area is cosmetic surgeries of female genitalia such as labia reduction, clitoral lift and vaginal tightening, all of which are very similar to the different types of FGM/C. Legal regulations concerning female genital cosmetic surgery were beyond the scope of this study. It is important though for future studies to explore the possible impact of not prohibiting female genital cosmetic surgeries on FGM/C.

Considering the magnitude and complexity of HCP duties in the prevention of FGM/C that comes in addition to their caregiving duties, comprehensive training of HCP is of utmost importance. Training is also expected to decrease medicalization [48]. Our study findings (Table 2) show that 24 countries (10 countries of origin and 14 countries of migration) conduct training for HCP. However, many studies have revealed a lack of knowledge among HCP on many aspects concerning FGM/C such as prevalence, identification of types, diagnosis and clinical management, as well as their professional roles and responsibilities, cultural competence, referral systems and legal issues [65, 77–81]. This could indicate that the reported training is inadequate in most of the above mentioned 24 countries. A possible explanation is that in as many as nine countries, of which seven are countries of migration; training is provided only on an ad-hoc basis.

Besides the competence of HCP, the availability of the necessary services is an important aspect of healthcare provision for those subjected to FGM/C. Infibulated girls and women can benefit from deinfibulation to improve health problems associated with infibulation and to allow sexual intercourse and childbirth [14, 18, 82–84]. Our findings (Table 4) show that deinfibulation is available in almost all responding countries. FGM/C is also associated with an increased risk of various psychological disorders in both children and adults [17, 85–87], challenges that seem to intensify in countries of migration [85, 88]. Cognitive behavioural therapy is the recommended management in such cases [18]. FGM/C is also associated with higher rates of sexual problems [16]. In countries of migration, experiences of stigma and/or being different can increase women’s concerns over their own sexuality [89–91]. The WHO recommends sexual counselling for preventing or treating female sexual dysfunction among women living with FGM/C, but not clitoral reconstruction [18]. Clitoral reconstruction was reported to be available in 13 countries (three countries of origin and 10 countries of migration) (Table 4). Many countries providing these services, however, combine surgical consultation with a multidisciplinary care model, including preoperative psycho-sexual counselling [92, 93]. Still, it is a cause of major concern that 17 countries, of which as many as nine countries are countries of origin, have reported a lack of psychological and sexual counselling services with a special competency on FGM/C (Table 4).

Another area for improvement is the systematic use of FGM/C codes in medical records. Records serve many central purposes such as providing an overview of the management of a particular disease, monitoring and improving quality of care, and providing robust databases for research [94]. Data on FGM/C in most medical records has so far been negligible for several reasons including lack of codes on FGM/C in some countries and inconsistencies in its use in others [32]. This is consistent with our findings (Table 5). Less than half of the participating countries reported to have designated medical codes on FGM/C, none of which are countries of origin. Only two countries (the Netherlands and UK) reported to use these codes systematically. Nevertheless, six of the countries that reported to have no designated medical code for FGM/C (Austria, Burkina Faso, Ghana, Kenya, Slovakia and Switzerland) reported during cross-checking to use ICD-10. Considering that in 2016 a new code on personal history of FGM/C (code Z91.7) was added to ICD-10 [59], all of these six countries could arguably be said to have available medical codes on FGM/C. These findings suggest that there is a great potential for strengthening healthcare provision and acquiring quality data on FGM/C prevalence and health complications, if countries were to adopt and systematically use ICD codes or harmonized national codes on FGM/C. Nonetheless, the mere introduction of ICD codes for FGM/C is not sufficient on its own, but has to be combined with comprehensive training on FGM/C. Existing research suggests that inconsistencies in the use of FGM/C codes are often attributed to a lack of knowledge, fear of causing stigmatization and concern over medical confidentiality [81, 95], and that coding improves considerably after training [95].

## Conclusion

For the last decades the international community has emphasised the importance of a multisectoral approach to tackle FGM/C and the crucial role of HCPs in this approach. Our study findings indicate that substantial progress has been made in the involvement of the health sector in both the treatment and prevention of FGM/C. There is nevertheless a need for countries of origin and countries of migration to incorporate systems for monitoring and evaluation into their plans of action. We have also identified five areas for improvement in the involvement of the health sector in the management of FGM/C: First there is a need to strengthen systematic training of HCP, particularly in countries of migration. Secondly, the role of HCP in averting and reporting FGM/C is unclear and inconsistent. There is therefore a need for studies that can evaluate and compare the different practices in different countries. It is also an ethical dilemma that HCP have a duty to avert and report in 19 and 16 countries respectively, but have a duty to provide relevant health education in only eight of these countries of which none are countries of origin. Thirdly, a stronger emphasis on the role of HCP in the provision of health education is needed everywhere but mainly in countries of origin. Further, provision of psychological and sexual counselling services with competency on FGM/C is lacking in 82% of the countries of origin and 42% of the countries of migration, and should be prioritized. Finally, routines and guidelines should be put in place to ensure the availability of FGM/C codes, particularly in countries of origin, and their systematic use in medical records in all countries.

## Additional file

Additional file 1: Check list – health policy on FGM/C (A blank copy of the questionnaire). (DOCX 91 kb)

## Abbreviations

EU: European Union
FGM/C: Female genital mutilation/cutting
HCP: Healthcare providers
ICD: International classification of diseases
NGO: non-governmental organizations
NSD: Norwegian Centre for Research Data
UN: United Nations
WHA: World Health Assembly
WHO: World Health Organization

## Footnotes

1 Targeted FGM/C practicing countries: Benin, Burkina Faso, Chad, Cote d’Ivoire, Djibouti, Egypt, Eritrea, Ethiopia, Gambia, Ghana, Guinea, Guinea Bissau, Indonesia, Iraq, Kenya, Liberia, Malaysia, Mali, Mauritania, Niger, Nigeria, Senegal, Sierra Leone, Somalia, Somaliland, Sudan, Thailand, Togo, Uganda, United Arab Emirates, Tanzania, Yemen. Targeted countries of migration: Austria, Australia, Belgium, Canada, Cyprus, Denmark, Estonia, Finland, France, Germany, Greece, Hungary, Ireland, Italy, Latvia, Lithuania, Netherlands, New Zealand, Norway, Slovakia, Saudi-Arabia, South Africa, Spain, Sweden, Switzerland, Portugal, UK, and the USA.

## References

[1] United Nations Children’s Fund. In: UNICEF, editor. Female genital mutilation/cutting: A global concern. New York; 2016.

[2] UNICEF (2014). Female Genital Mutilation/cutting: what might the future hold?.

[3] van Baelen L, Ortensi L, Leye E (2016). Estimates of first-generation women and girls with female genital mutilation in the European Union, Norway and Switzerland. Eur J Contracept Reprod Health Care.

[4] Leye E, et al. Towards a better estimation of prevalence of female genital mutilation in the European Union: interpreting existing evidence in all EU member states. Genus. 2014;70(1)

[5] Macfarlane A, Dorkenoo E (2015). Prevalence of female genital mutilation in England and Wales: National and local estimates.

[6] Ziyada MM, Norberg-Schulz M, Johansen REB (2016). Estimating the magnitude of female genital mutilation/cutting in Norway: an extrapolation model. BMC Public Health.

[7] Goldberg H (2016). Female genital mutilation/cutting in the United States: updated estimates of women and girls at risk, 2012. Public Health Rep.

[8] Hearst AA, Molnar AM. Female genital cutting: an evidence-based approach to clinical management for the primary care physician. Amsterdam: Elsevier. Mayo Clin Proc. 2013;88(6):618–29. 10.1016/j.mayocp.2013.04.004. 10.1016/j.mayocp.2013.04.004 23726401

[9] Dawson A (2015). Evidence to inform education, training and supportive work environments for midwives involved in the care of women with female genital mutilation: a review of global experience. Midwifery.

[10] Higginbottom GM (2013). “I have to do what I believe”: Sudanese women’s beliefs and resistance to hegemonic practices at home and during experiences of maternity care in Canada. BMC Pregnancy Childbirth.

[11] World Health Organization (2008). Eliminating female genital mutilation: An interagency statement–OHCHR, UNAIDS, UNDP, UNECA.

[12] UNICEF (2013). Female Genital Mutilation/Cutting: A statistical overview and exploration of the dynamics of change.

[13] Berg RC, Underland V. Immediate health consequences of female genital mutilation/cutting (FGM/C). Systematic Review, vol. 8: Kunnskapssenteret; 2014.

[14] Berg RC (2014). Effects of female genital cutting on physical health outcomes: a systematic review and meta-analysis. BMJ Open.

[15] WHO Study Group on Female Genital Mutilation and Obstetric Outcome (2006). Female genital mutilation and obstetric outcome: WHO collaborative prospective study in six African countries. Lancet.

[16] Berg RC, Denison E (2012). Does female genital mutilation/cutting (FGM/C) affect women’s sexual functioning? A systematic review of the sexual consequences of FGM/C. Sexuality Research and Social Policy.

[17] Berg RC, Denison E, Fretheim A (2010). Psychological, social and sexual consequences of female genital mutilation/cutting (FGM/C): A systematic review of quantitiative studies, 12/2010.

[18] World Health Organization (2016). WHO guidelines on the management of health complications from female genital mutilation.

[19] WHO, Traditional practices affecting the health of women and children: female circumcision, childhood marriage, nutritional taboos, etc.: report of a seminar, Khartoum, 10–15 February, 1979. WHO/EMRO technical publication; no. 2. 1979, Alexandria [Egypt]: World Health Organization, Regional Office for the Eastern Mediterranean. 2 v.

[20] WHO and UNICEF. Female genital mutilation: a joint WHO/UNICEF/UNFPA statement: World Health Organization; 1997.

[21] WHO (2001). Female Genital Mutilation. The prevention and the management of the health complicataions. Policy guidelines for nurses and midwives.

[22] WHO (2001). Management of pregnancy, childbirth and the postpartum period in the presence of female genital mutilation.

[23] WHO (2001). Female genital mutilation - a teacher’s guide.

[24] WHO (2001). Female genital mutilation - A student’s manual. Integrating the preveition and the management of the health complications into the curricula of nursing and midwifery.

[25] UN General Assembly. Intensifying global efforts for the elimination of female genital mutilations, vol. 3: UN GA, A/C; 2012. p. 67.

[26] WHA. Female genital mutilation. Agenda item 11.8 24 May 2008. 61’thWHA W.H. Assembly. Geneva: World Health Organization. p. 2008.

[27] FGM Prevention Programme - Department of Health (2015). FGM prevention programme - understanding the FGM enhanced dataset - updated guidance and clarification to support implementation.

[28] Department of Health (2015). Female genital mutilation risk and safeguarding.

[29] Home Office UK. Mandatory reporting of female genital mutilation - procedural information: Home office United Kingdom; 2015.

[30] United Kingdom Government. Multi-agency practice guidelines*:* Female Genital Mutilation. UK; 2011.

[31] BLD og HOD (2008). Veileder om regelverk, roller og ansvar knyttet til kjønnslemlestelse.

[32] European Institute for Gender Equality. Female genital mutilation in the European Union and Croatia: EIGE; 2013.

[33] Statens Helsetilsyn. In: Helsetilsyn S, editor. Veileder for helsepersonell i Norge om kvinnelig omskjæring: Statens Helsetilsyn; 2000.

[34] The Royal Australian College of Obstetricians and Gynaecologists. FGM- information for Australian health professionals. Sydney; 1997.

[35] Nelson E, Pierce M, Robertson D, Simmonds A (2013). Female genital cutting - clinical practical guidelines. J Obstet Gynaecol Can.

[36] Royal College of obstetricians & Gynaecologists (2015). Female genital mutilation and its management.

[37] University of Nairobi, M.o.H (2008). Management of complications pregnancy, childbirth and the postpartum period in the presence of FGM/C- A reference manual for health service provider.

[38] Nour NM (2004). Female Genital Cutting: Clinical and Cultural Guidelines. Obstet Gynecol Surv.

[39] Johnson C, Nour NM (2007). Surgical techniques: Defibulation of type III female genital cutting. J Sex Med.

[40] Kaplan A, Hechavarría ST, Puppo NLA. Manual for the Managemenet and Prevention of FGM/C for Health Professionals in The Gambia: Wassu-UAB Foundation; 2016. p. 69.

[41] Muthumbi J (2015). Female genital mutilation: a literature review of the current status of legislation and policies in 27 African countries and Yemen. Afr J Reprod Health.

[42] Leye E, Deblonde J, Temmerman M (2004). A comparative analysis of the different legal approaches in the 15 EU member states, and the respective judicial outcomes in Belgium, France, Spain, Sweden and the UK.

[43] WHO (2010). WHA progress report.

[44] Johansen REB (2013). What works and what does not: a discussion of popular approaches for the abandonment of female genital mutilation. Obstet Gynecol Int.

[45] Population Reference Bureau (2001). Abandoning female genital cutting. Prevalence, Attitudes, and Efforts to End the Practice.

[46] Berg RC, Denison E. Interventions to reduce the prevalence of female genital mutilation/cutting in African countries. Campbell Syst Rev. 2012;8(9)

[47] UNFPA (2010). Global strategy to stop health-care providers from performing female genital mutiliation.

[48] World Health Organization (2016). WHO guidelines on the management of health complications from female genital mutilation: policy brief. WHO guidelines on the management of health complications from female genital mutilation: policy brief.

[49] Barne- og likestillingsdepartementet and Helse- og omsorgsdepartementet (2009). Veileder om regelverk, roller og ansvar knyttet til kjønnslemlestelse.

[50] Costello S (2015). Female genital mutilation/cutting: risk management and strategies for social workers and health care professionals. Risk Manag Healthc Policy.

[51] Shell-Duncan B, Njue C, Moore Z (2017). The Medicalization of Female Genital Mutilation/Cutting: What do the Data Reveal. Evidence to End FGM/C: Research to Help Women Thrive.

[52] Berggren V (2004). An explorative study of Sudanese midwives’ motives, perceptions and experiences of re-infibulatino after birth. Midwifery.

[53] Almroth-Berggren V (2001). Reinfibulation among women in a rural area in Central Sudan. Health Care Women Int.

[54] Serour GI (2010). The issue of reinfibulation. Int J Gynecol Obstet.

[55] Paliwal P (2014). Management of type III female genital mutilation in Birmingham, UK: a retrospective audit. Midwifery.

[56] Abdulcadir J, Rodriguez MI, Say L (2015). A systematic review of the evidence on clitoral reconstruction after female genital mutilation/cutting. Int J Gynecol Obstet.

[57] Foldes P, Louis-Sylvestre C (2006). Résultats de la réparation chirurgicale du clitoris après mutilation sexuelle: 453 cas. Gynécol Obstét Fertil.

[58] Foldès P, Cuzin B, Andro A (2012). Reconstructive surgery after female genital mutilation: a prospective cohort study. Lancet.

[59] WHO (2016). International Classification of Diseases.

[60] UNFPA UNICEF. Accelerating change by the numbers. “016 annual report of the UNFPA-UNICEF joint Programme on female genital mutilation/cutting: accelerating change”: UNFPA UNICEF; 2016.

[61] Ali A. Knowledge and attitudes of female genital mutilation among midwives in eastern Sudan. Reprod Health J. 2012; https://doi.org/10.1186/1742-4755-9-23. 10.1186/1742-4755-9-23 PMC3499224 23020897

[62] Refaat A (2009). Medicalization of female genital cutting in Egypt. East Mediterr Health J.

[63] Njue C, Askew I (2004). Medicalization of female genital cutting among the Abagusii in Nyanza Province, Kenya.

[64] Kaplan A (2013). Knowledge, attitudes and practices of female genital mutilation/cutting among health care professionals in the Gambia: a multiethnic study. BMC Public Health.

[65] Cappon S, et al. Female genital mutilation: knowledge, attitude and practices of Flemish midwives. Midwifery. 2014 10.1016/j.midw.2014.11.012 25575861

[66] Allhoff F, Group, I.D.L.W (2008). Introduction. Physicians at War: The Dual-Loyalties Challenge.

[67] Group, I.D.-L.W. Dual-loyalty and human rights in health professional practice: proposed guidelines and institutional mechanisms. In: Physicians at War: Springer; 2008. p. 15–38. 10.1007/978-1-4020-6912-3_2.

[68] Hodes D (2016). Female genital mutilation in children presenting to a London safeguarding clinic: a case series. Arch Dis Child.

[69] Leye E, Middelburg A. Female genital mutilation in Europe from a child right’s perspective: Routledge international handbook of Children’s rights Studies; 2015. p. 295.

[70] Lien I-L. Prosecution of the offence of female genital mutilation/cutting in Norway. Int J Law Policy Fam. 2017;

[71] Creighton SM (2016). Multidisciplinary approach to the management of children with female genital mutilation (FGM) or suspected FGM: service description and case series. BMJ Open.

[72] Shell-Duncan B (2001). The medicalization of female “circumcision”: harm reduction or promotion of a dangerous practice?. Soc Sci Med.

[73] Boddy JP (2007). Civilizing women: British crusades in colonial Sudan.

[74] Serour G (2013). Medicalization of female genital mutilation/cutting. Afr J Urol.

[75] Doucet M-H, Pallitto C, Groleau D (2017). Understanding the motivations of health-care providers in performing female genital mutilation: an integrative review of the literature. Reprod Health.

[76] Leye E (2007). An analysis of the implementation of laws with regard to female genital mutilation in Europe. Crime Law Soc Chang.

[77] Abdulcadir J, Rodriguez M, Say L. Research gaps in the care of women with female genital mutilation: an analysis. BJOG Int J Obstet Gynaecol. 2014; 10.1111/1471-0528.13217 25514892

[78] Turkmani S, et al. A survey of Australian midwives’ knowledge, experience, and training needs in relation to female genital mutilation. Women Birth. 2017; 10.1016/j.wombi.2017.06.009 28687260

[79] Leye E (2008). Female genital mutilation: knowledge, attitudes and practices of Flemish gynaecologists. Eur J Contracept Reprod Health Care.

[80] Dawson A (2015). Midwives’ experiences of caring for women with female genital mutilation: insights and ways forward for practice in Australia. Women Birth.

[81] Johansen REB (2006). Care for infibulated women giving birth in Norway: an anthropological analysis of health workers’ management of a medically and culturally unfamiliar issue. Med Anthropol Q.

[82] Johansen REB (2016). Undoing female genital cutting: Perceptions and experiences of infibulation, defibulation and virginity among Somali and Sudanese migrants in Norway. Cult Health Sex.

[83] Johansen REB. Virility, pleasure and female genital cutting. Perceptions and experiences of medicalized defibulation among Somali and Sudanese migrants in Norway. Reprod Health. 2017; 10.1186/s12978-017-0287-4 PMC5303310 28187741

[84] Nour NM, Michels KB, Bryant AE (2006). Defibulation to treat female genital cutting: effect on symptoms and sexual function. Obstet Gynecol.

[85] Vloeberghs E (2012). Coping and chronic psychosocial consequences of female genital mutilation in the Netherlands. Ethn Health.

[86] Kizilhan JI (2011). Impact of psychological disorders after female genital mutilation among Kurdish girls in Northern Iraq. Eur J Psychiatr.

[87] Behrendt A, Moritz S (2005). Posttraumatic stress disorder and memory problems after female genital mutilation. Am J Psychiatr.

[88] Johansen REB (2002). Pain as a counterpoint to culture: toward an analysis of pain associated with infibulation among Somali immigrants in Norway. Med Anthropol Q.

[89] Johnsdotter S, Essén B (2015). Culture and sexual scripts in and out of Africa*:* Understanding FGC in relation to sexuality. Management of women with FGM/C: 1st international consultation, university Paris-1 Pantheon Sorbonne & School for advanced studies in social sciences, France, 27–28 January, 2015.

[90] Johansen REB, Hernlund Y, Shell-Duncan B (2007). Experiencing sex in exile—can genitals change their gender?. Transcultural bodies: female genital cutting in global context.

[91] Ahmadu F, Herlund Y, Shell-Duncan B (2007). Ain’t I a woman, too? Challenging myths of sexual dysfunction of circumcised women. Transcultural bodies: Female genital cutting in global context.

[92] De Schrijver L, Leye E, Merckx M. A multidisciplinary approach to clitoral reconstruction after female genital mutilation: the crucial role of counselling. Eur J Contracept Reprod Health Care. 2016:1–7. 10.3109/13625187.2016.1172063 27111038

[93] Antonetti NE, Fall S, Beltran L (2015). Benefits of multidisciplinary care for excised women. J Gynecol Obstet Biol Reprod (Paris).

[94] Arts DG, De Keizer NF, Scheffer G-J (2002). Defining and improving data quality in medical registries: a literature review, case study, and generic framework. J Am Med Inform Assoc.

[95] International Centre for Reproductive Health/Centre Hospitalier Universitaire Saint Pierre. Project voor de registratie van gevallen van vrouwelijke genital verminking in ziekenhuizen. Ghent; 2014.

