# Supplementary material for: Health sector involvement in the management of female genital mutilation/cutting in 30 countries
Source: BMC Health Serv Res. 2018 Apr 4;18:240. doi: 10.1186/s12913-018-3033-x (PMC5883890; doi:10.1186/s12913-018-3033-x)
Supplement: Supplementary file 1 — Check list – health policy on FGM/C (A blank copy of the questionnaire). (DOCX 91 kb) [file 12913_2018_3033_MOESM1_ESM.docx]

**Health policy on female genital mutilation (FGM): decisions, plans, and actions endorsed by the government to achieve specific health care goals relating to the outlined six policy areas.**

**Name of country:** Click here to enter text.

| **Policy area** | **Policy area detail** | **National policy** | **Type of policy document** |
| --- | --- | --- | --- |
| **Training/capacity building** | Training of health professionals in FGM |   If yes, where is it included?  In standard medical curriculum  In standard specialist curriculum  In standard nursing curriculum  In standard midwifery curriculum  Other, specify: Click here to enter text.  Also if yes, what are the covered training areas?  Recognition of different types of FGM and its related complications  Coding systems related to FGM  Management of complications related to FGM  Cultural competence  Other, specify: Click here to enter text. | National plan of action  Professional guidelines  Official guidelines  Laws and legislations |
|  |  |  |  |
| **Prevention** | Provision of information to individuals and families by health professionals during consultations | Required to provide information  Not required, but encouraged  Not a requirement | National plan of action  Professional guidelines  Official guidelines  Laws and legislations |
|  |  |  |  |
| **Medicalization** | Perform FGM on minors | Illegal and not performed  Illegal, but performed  Legal, but discouraged  Legal and encouraged  No regulation or policy | National plan of action  Professional guidelines  Official guidelines  Laws and legislations |
|  |  |  |  |
|  | Perform FGM on adult | Illegal and not performed  Illegal, but performed  Legal, but discouraged  Legal and encouraged  No regulation or policy Other, specify: Click here to enter text. | National plan of action  Professional guidelines  Official guidelines  Laws and legislations |
|  |  |  |  |
|  | Perform re-infibulation | Illegal and not performed  Illegal, but performed  Legal, but discouraged  Legal and encouraged  No regulation or policy | National plan of action  Professional guidelines  Official guidelines  Laws and legislations |
|  |  |  |  |
| **Health care provision** | Defibulation | Available  Not available  If available,  Public health services  Private health services  And:  Specialized  Mainstreamed | National plan of action  Professional guidelines  Official guidelines |
|  |  |  |  |
|  | Psychological counselling | Available  Not available  If available,  Public health services  Private health services  And:  Specialized  Mainstreamed | National plan of action  Professional guidelines  Official guidelines |
|  |  |  |  |
|  | Sexual counselling | Available  Not available  If available,  Public health services  Private health services  And:  Specialized  Mainstreamed | National plan of action  Professional guidelines  Official guidelines |
|  |  |  |  |
|  | Clitoral reconstruction | Available  Not available  If available,  Public health services  Private health services  And:  Specialized  Mainstreamed | National plan of action  Professional guidelines  Official guidelines |
|  |  |  |  |
|  | Other clinical management of complications, specify:  Click here to enter text. | Available  Not available  If available,  Public health services  Private health services  And:  Specialized  Mainstreamed | National plan of action  Professional guidelines  Official guidelines |
|  |  |  |  |
| **Registration in health/medical registries** | Availability and use of medical codes | Available and used systematically  Available, but not systematically used  Not available  If codes are available (regardless of being systematically used or not), please specify what types of codes. You can check more than one box.  Diagnostic, FGM status  Diagnostic, FGM complications  Procedural, FGM management  Other, specify:  Click here to enter text. | National plan of action  Professional guidelines  Official guidelines |
|  |  |  |  |
| **Reporting to legal authorities** | Reporting of planned FGM | Duty to avert take precedence over code of silence  Code of silence take precedence over duty to avert | National plan of action  Professional guidelines  Official guidelines  Laws and legislations |
|  |  |  |  |
|  | Reporting performed FGM | Duty to report take precedence over code of silence  Code of silence take precedence over duty to report | National plan of action  Professional guidelines  Official guidelines  Laws and legislations |
|  |  |  |  |

**Policy implementation**

| **Level of implementation** | **Financing** | **Accountability** | **Monitoring and evaluation** |
| --- | --- | --- | --- |
| Fully implemented  Partially implemented  Not implemented  Do not know | Allocation of budget to the national action plan/health policy on FGM:  Budget allocated and earmarked  Budget allocated, but not earmarked  Budget not allocated | Coordination bodies are assigned  No Coordination bodies are assigned | Monitoring and evaluation systems in place  Monitoring and evaluation systems not in place |
